# Identification and Targeted Correction of a Pathogenic *PMP22* Deep Intronic Variant

**DOI:** 10.3390/ijms27083572

**Published:** 2026-04-16

**Authors:** Polina Chausova, Aysylu Murtazina, Igor Bychkov, Inga Anisimova, Alexandra Ilyushkina, Kamilla Mollaeva, Asiyat Magomedova, Vyacheslav Tabakov, Tatyana Hegay, Alena Chukhrova, Aleksandr Polyakov

**Affiliations:** 1 Research Centre for Medical Genetics, Moscow 115522, Russia; aysylumurtazina@gmail.com (A.M.); bychkov@med-gen.ru (I.B.); anisimova-inga@med-gen.ru (I.A.); alexilyuskina@gmail.com (A.I.); achukhrova@yandex.ru (A.C.); apol@dnalab.ru (A.P.); 2 Neuromed Medical Center, Machachkala 367000, Russia; t.mollaev75@mail.ru; 3 State Budgetary Institution of the Republic of Dagestan “Republican Diagnostic Center”, Machachkala 367000, Russia; chocolate05@gmail.com; 4 Medical Center “Lekar”, Machachkala 367015, Russia; 5 Immunology and Human Genomics of the Academy of Science of the Republic of Uzbekistan, Tashkent 100060, Uzbekistan; trhegay@gmail.com

**Keywords:** *PMP22*, DSS, HNPP, hereditary peripheral demyelinating neuropathies

## Abstract

Pathogenic variants in the *PMP22* gene can lead to hereditary peripheral demyelinating neuropathies of varying severity, including hereditary neuropathy with liability to pressure palsies (HNPP), Charcot–Marie–Tooth disease types 1A and 1E (CMT1A, CMT1E), Roussy–Lévy syndrome, and Dejerine–Sottas disease (DSS). This study describes a novel deep intronic variant c.179-2809A>G in the *PMP22* gene, identified in two unrelated Avar families from Dagestan republic, Russia. This variant was identified in nine patients. In seven cases, it was detected in a heterozygous state, leading to the development of HNPP. In two cases, this variant was found in a homozygous state, resulting in a more severe CMT1A phenotype (Dejerine–Sottas disease). The performed functional analysis allowed us to characterize the deleterious effect of this variant and propose an approach for personalized antisense therapy. This work demonstrates that, in Avar people with HNPP traits, variant c.179-2809A>G should be considered as disease-causing and included in standard genetic testing.

## 1. Introduction

Hereditary peripheral demyelinating neuropathies represent a heterogeneous group of inherited neuromuscular disorders characterized by damage to the myelin sheaths of peripheral nerves. The most common cause of peripheral neuropathy is the presence of pathogenic or likely pathogenic variants in the *PMP22* gene. The PMP22 protein is primarily abundant in Schwann cells of the peripheral nervous system and functions as an integral membrane glycoprotein of compact myelin, consisting of 160 amino acid residues [1,2,3,4,5]. PMP22 accounts for approximately 2–5% of all proteins in compact myelin [6,7,8]. It plays a crucial role in the formation and maintenance of compact myelin and modulates Schwann cell proliferation and apoptosis [7].

Different types of mutations in the *PMP22* gene can lead to distinct phenotypes. The most common type of mutation is a 1.5 Mb duplication of a chromosome 17 segment (17p11.2–12), which results in the phenotype of Charcot–Marie–Tooth disease type 1A (CMT1A, OMIM_118220). CMT1A is characterized by muscle weakness and atrophy, reduced reflexes, distal sensory impairment, deformities of the feet and hands, decreased nerve conduction velocity (NCV), and hypertrophic segmental demyelination and remyelination, which are pathologically manifested as onion bulb formations [6]. Triplication of this chromosomal segment results in a more severe phenotype [7,9]. A gross deletion of the 17p11.2–12 segment leads to hereditary neuropathy with liability to pressure palsies (HNPP, OMIM_162500), which is characterized by episodic, recurrent sensory and motor mononeuropathies at sites of nerve compression. Histological and electrophysiological studies reveal myelin abnormalities, including loss of myelinated fibers, thin myelin sheaths, demyelination and remyelination, prominent tomacula formation, and occasional onion bulb structures [10]. Point mutations in the *PMP22* gene are identified in a smaller number of patients with *PMP22*-associated neuropathies. Heterozygous variants leading to loss of protein function (LoF) most commonly result in HNPP, whereas missense variants are more frequently associated with more severe forms, such as CMT1A and CMT1E. (OMIM_118300) [6,11]. Most missense variants are observed in *PMP22*-associated neuropathies with an autosomal dominant (AD) mode of inheritance.

Also, different types of nucleotide sequence variant can lead to Dejerine–Sottas syndrome (DSS, OMIM_145900), which is characterized by onset at birth or in early childhood, delayed motor development, generalized hypotonia, and very slow NCV [6]. As of the current version of the Human Genetic Mutation Database (HGMD Professional, 2025.1, http://www.hgmd.cf.ac.uk/ac, accessed on 15 February 2026), more than 37 pathogenic variants associated with DSS have been reported. These include gross deletions of the 17p11.2–12 segment, gross duplications of the 17p11.2–12 segment, splice-affecting variants, deletions of exons 2–3 or exon 5, a variant leading to loss of the stop codon resulting in a protein elongated by nine amino acid residues, and different missense variants. Among these, only seven variants have been reported in homozygous or compound heterozygous states.

Currently, treatment of *PMP22*-associated neuropathies is symptomatic; however, active research is ongoing in the field of targeted therapy development [12,13]. The most common therapeutic strategy focuses on suppressing *PMP22* overexpression resulting from an increased gene copy number.

The Avar ethnic group is relatively isolated and resides mostly in the Republic of Dagestan, Russia. It comprises approximately 1.3 million people worldwide, of whom around 1 million reside in Russia. In this study, we present clinical, electrophysiological, and molecular genetic analyses of two families from Dagestan, of Avar origin, whose affected members have DSS and HNPP phenotypes. These individuals carry a likely pathogenic deep intronic variant in the homozygous or heterozygous state, which leads to the activation of a pseudoexon within intron 3. We also demonstrate that this pseudoexon may represent a potential target for splice-modulating therapy.

## 2. Results

### 2.1. Clinical Data

**Figure 1:**
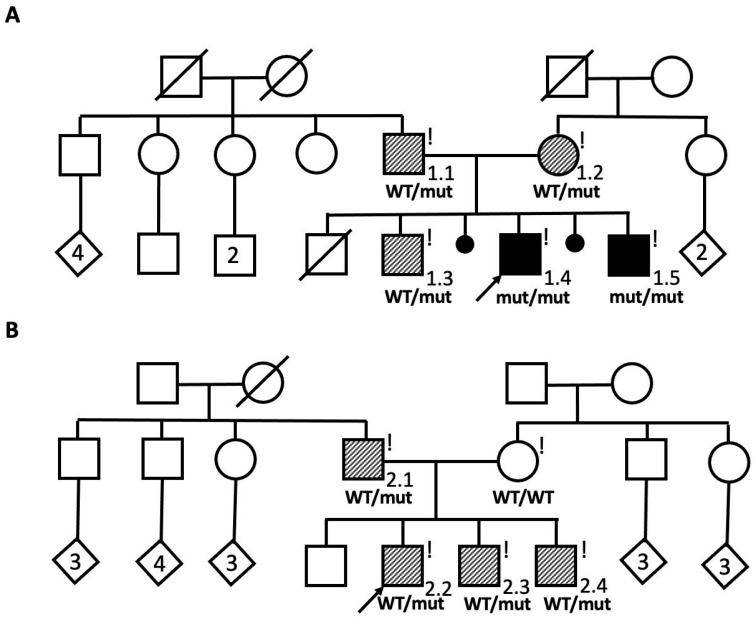
The pedigrees of two described families. (**A**) The pedigree of Family 1. Solid black symbols represent the affected siblings with the Dejerine–Sottas phenotype, while striped symbols indicate the probands’ parents and older brother, affected with HNPP. Exclamation marks denote individuals who underwent clinical examination. (**B**) The pedigree of Family 2. Striped symbols indicate the proband, proband’s father, and younger brothers, affected with HNPP. Exclamation marks denote individuals who underwent clinical examination. The diamond symbols indicate individuals of unspecified sex, with the numbers inside the symbols representing the number of siblings. WT—wild type; mut—pathogenic variant.

Family 1:

A non-consanguineous Avar family with two affected male siblings (Patients 1.4 and 1.5) were referred to the genetic center with diagnosis of hereditary neuropathy (Figure 1A).

Both affected siblings were non-ambulant due to severe leg muscle weakness and atrophy by the ages of 4 and 10 years, respectively. They were born at term with uncomplicated deliveries, normal birth length and body weight. Since birth, they exhibited generalized muscle hypotonia and poor suckling. Delayed motor development was noted from the first months of life: they held their heads up at 9 months, sat independently at 15 months, and stood with support from 20 months. However, neither patient ever achieved independent walking. Intellectual development was age-appropriate.

**Figure 2:**
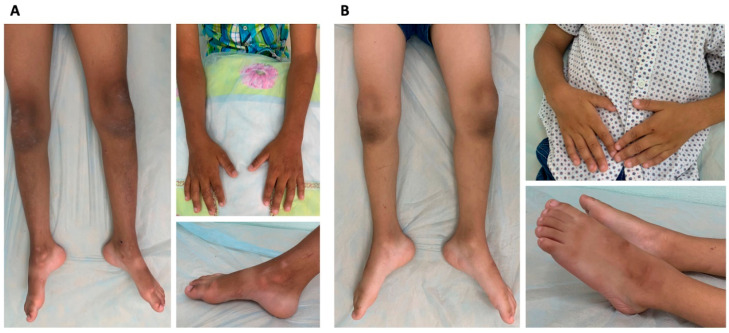
Clinical presentations of the affected siblings with DSS from the first family: the older brother (Patient 1.4 in Figure 1A) (**A**) and younger brother (Patient 1.5 in Figure 1A) (**B**). Both patients exhibited diffuse muscle atrophy, predominantly affecting the lower leg and foot muscles.

During clinical examination, both siblings exhibited generalized limb muscle hypotonia, predominantly distal muscle atrophy, muscle weakness (ranging from plegia in distal muscles to 2–3/5 on the MRC scale in proximal muscles), generalized areflexia, and a positive Gower’s sign (Figure 2A,B). No significant sensory disturbances were observed, and cranial nerve functions, including hearing, were normal.

Nerve conduction study (NCS) in the older sibling revealed the absence of compound muscle action potentials (CMAPs) and sensory nerve action potentials (SNAPs) in both the upper and lower limbs. In the younger brother, only ulnar CMAPs were detected, with amplitudes of 0.6 mV and 1.1 mV (normal range > 6.0 mV) and distal latencies of 9.1 ms and 6.5 ms (normal range < 3.3 ms) on the right and left sides, respectively. No other CMAPs or SNAPs were observed. Nerve US demonstrated a diffuse increase in the cross-sectional area (CSA) in both children, consistent with generalized demyelinating neuropathy.

Both parents (Patients 1.1 and 1.2 in Figure 1A), who considered themselves healthy, had experienced frequent episodes of numbness and tingling in the upper and lower extremities.

**Figure 3:**
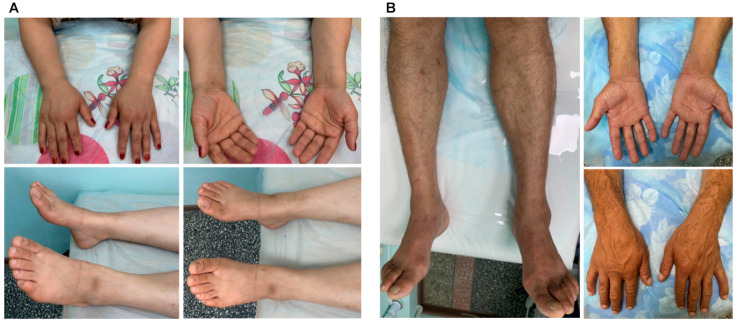
The clinical presentations of the affected parents with HNPP from the first family. The mother (Patient 1.2 in Figure 1A) exhibited mild hypotrophy of the foot and lower arm muscles bilaterally (**A**). Clinical examination of the father (Patient 1.1 in Figure 1A) revealed atrophy of the lower leg and foot muscles, as well as mild hypotrophy of the left hypothenar muscles (**B**).

Clinical examination of the proband’s mother (Patient 1.2) revealed mild hypotrophy of the foot muscles and reduced ankle tendon reflexes (Figure 3A, Table 1).

The father (Patient 1.1) exhibited atrophy of the lower leg and foot muscles, mild hypotrophy of the left hypothenar muscles, and reduced brachioradialis and ankle tendon reflexes (Figure 3B, Table 1).

NCS in the mother showed signs of local demyelinating changes in the left ulnar nerve at the elbow, the left median nerve at the wrist, and demyelinating peroneal neuropathy at the fibular head. NCS in the father revealed more pronounced changes, including electrophysiological signs of local demyelinating neuropathy in the ulnar and median nerves bilaterally, as well as diffuse secondary axonal neuropathy in the lower limbs. Nerve US demonstrated a localized increase in the CSA of the median, ulnar, and peroneal nerves at entrapment sites in both parents.

The probands’ older brother (Patient 1.3 in Figure 1A), who was previously considered unaffected, began experiencing periodic episodes of hand numbness at the age of 14. NCS revealed bilateral conduction blocks in the ulnar nerve at the elbow, consistent with multifocal demyelinating neuropathy.

A clinical diagnosis of hereditary peripheral demyelinating neuropathies was made for both siblings (Patients 1.4 and 1.5) based on the data obtained, including NCS data. The clinical diagnosis of HNPP was made for their parents (Patients 1.1 and 1.2) and older brother (Patient 1.3).

Family 2:

The proband was an affected male from a non-consanguineous Avar family (Figure 1B, Patient 2.2). He had experienced frequent episodes of numbness and tingling in the hands and an episode of paresis. Two family members (Patients 2.1 and 2.3) had histories of a single episode of right arm paresis, both with full recovery. During clinical examination, all affected individuals exhibited only reduced tendon reflexes, while NCS revealed typical electrophysiological features of HNPP in every patient (Table 1).

### 2.2. Molecular Genetic Analysis

Family 1

DNA samples from the siblings were tested for large deletions and duplications in the *PMP22* gene by quantitative MLPA method. As a result of the analysis, no large deletions or duplications were identified. Subsequently, the genes responsible for the development of peripheral neuropathies were analyzed in the older sibling using the virtual «Neuropathies» panel based on WGS. The previously undescribed nucleotide sequence variant c.179-2809A>G was identified within intron 3 of the *PMP22* gene in a homozygous/hemizygous state (the coverage depth at this site was ×23). The identified variant was not registered in the Genome Aggregation Database (gnomAD v3.1.2, https://gnomad.broadinstitute.org/, accessed on 15 February 2026). The splicing impact prediction algorithm SpliceAI evaluated this variant as highly spliceogenic (Delta score–0.74), while Squirls and Spip classified it as neutral (Scores 0.03 and 0.26, respectively). During segregation analysis by Sanger sequencing, the variant was identified in homozygous state in the siblings and in the heterozygous state in their parents and their older brother (Figure 1A). According to the ACMG criteria, the variant was classified as a variant of uncertain clinical significance (PM2. PP1, PP4).

Family 2

Considering the data obtained from the study of Family 1 and the ethnic background of the members of Family 2, the proband from Family 2 was directly tested for the c.179-2809A>G variant in the *PMP22* gene using Sanger sequencing (A previous genetic test did not reveal a *PMP22* deletion). As a result of the analysis, this variant was identified in a heterozygous state. Segregation analysis detected the variant in a heterozygous state in the proband’s two brothers and father (Figure 1B).

### 2.3. RNA Analysis of the c.179-2809A>G Variant

**Figure 4:**
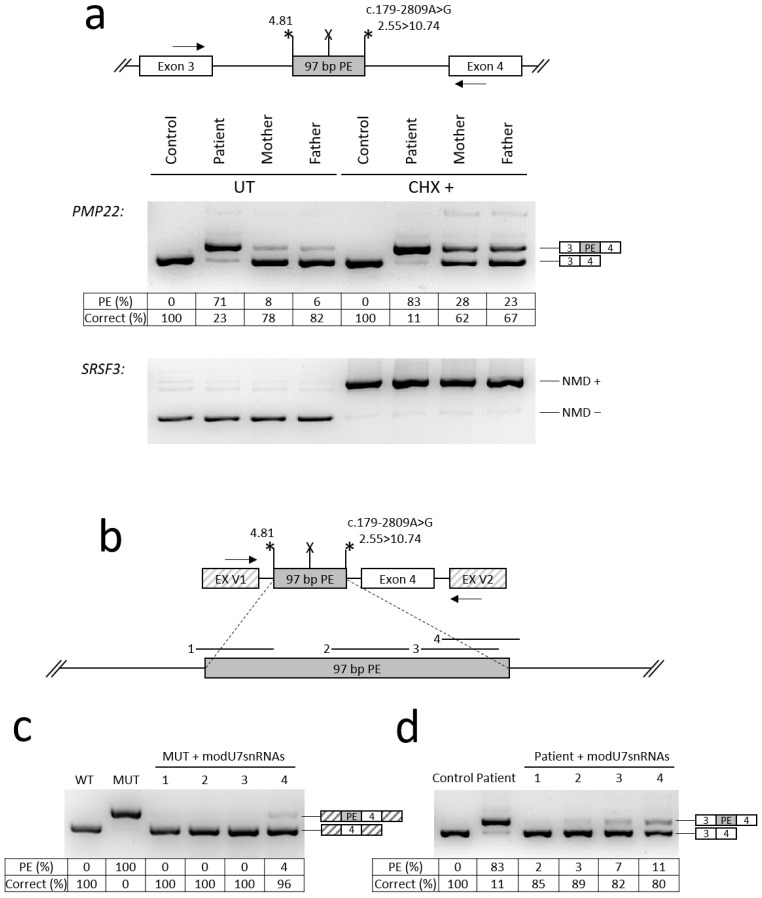
Functional analysis of the c.179-2809A>G variant and testing of antisense splice-modulating molecules. The mean amounts of wild-type (WT) and PE-containing (PE) mRNA isoforms were calculated by fragment analysis of PCR products of biological replicates (the amounts of minor isoforms are not shown). Asterisks indicate PE’s splice site strength (MaxEntScan score); X—premature stop codon; arrows—locations of PCR primers. (**a**) Analysis of mRNA from patient’s fibroblasts (Patient–Patient 1.4, mother—Patient 1.2, father—Patient 1.1, all in Figure 1A). *SRSF3* mRNA isoforms represent the control for NMD inhibition. «NMD +» isoform carries a premature stop codon and, in the absence of CHX, efficiently degrades by NMD. «NMD −» is the NMD-insensitive isoform. (**b**) The structure of minigene and location of targets for modU7snRNAs 1–4. (**c**) Results of the minigene and modU7snRNA co-transfection experiments. WT—wild-type minigene; MUT—minigene with the c.179-2809A>G variant; 1-4–modU7snRNAs from panel (**b**). (**d**) Results of the transduction of patient’s fibroblasts with lentiviral vectors expressing modU7snRNAs.

To establish the effect of c.179-2809A>G on *PMP22* splicing, we obtained fibroblast cultures of the older sibling and his parents from Family 1. The c.179-2809A>G variant was predicted to create a strong donor splice site in *PMP22* intron 3, which could potentially lead to pseudoexon (PE) activation. To verify this PE, we performed the analysis of RNA extracted from cultured fibroblasts of the patient (Patient 1.4 in Figure 1A) and his parents (mother—Patient 1.2, father—Patient 1.1, both in Figure 1A). To better represent the ratio of isoforms, we treated fibroblasts with cycloheximide (CHX), the inhibitor of nonsense-mediated mRNA decay (NMD) pathway. The results of RNA analysis revealed the presence of a 97 bp PE and a residual amount of the wild-type mRNA isoform in the patient’s sample (Figure 4). The identified PE leads to premature stop codon formation.

Considering all collected data (clinical examination, instrumental studies, genetic testing, and functional analysis), the variant c.179-2809A>G has been reclassified as likely pathogenic (PVS1_Strong, PM2, PP1, PP4).

### 2.4. Testing of Antisense Molecules Against PE in a Minigene Assay and in Patient’s Cultured Fibroblasts

To investigate approaches to personalized antisense-based therapy for the c.179-2809A>G variant by blocking the identified PE, we designed a minigene encompassing the studied genomic locus (Figure 4b). The results of transfection of HEK293T cells with the minigene demonstrated the same splicing pattern insertion of 97 bp PE (Figure 4c). Next, we designed various antisense sequences targeting PE splice sites or splicing enhancer motifs and incorporated them into modified U7 small nuclear RNAs (modU7snRNAs) (Figure 4c). All four modU7snRNAs efficiently blocked the PE, leading to almost complete restoration of the correct mRNA isoform (Figure 4c).

To test antisense molecules in the patient’s cultured fibroblasts, modU7snRNA cassettes were cloned into lentiviral vectors. Fibroblasts were transduced with lentiviral particles at MOI ~ 5 and, after 24 h, subjected to puromycin selection for 4 days. Analysis of fibroblast RNA demonstrated data similar to the minigene assay: all modU7snRNAs almost completely blocked the inclusion of the PE, with the most effective modU7snRNA targeting PE’s acceptor splice site (Figure 4d).

## 3. Discussion

Pathogenic and likely pathogenic variants in the *PMP22* gene can lead to phenotypes of varying severity, ranging from the milder form of HNPP to the more severe DSS. According to literary (HGMD Professional 2025.1), only one case of a pathogenic non-canonical splicing variant in the *PMP22* gene, c.79-13T>A has been identified [14]. And only two cases have been reported when the variant in the family in heterozygous statesled to HNPP, while in the homozygous state it resulted in Dejerine–Sottas syndrome (DSS) [15,16]. We described cases in which the deep intronic variant c.179-2809A>G, when homozygous, led to the DSS phenotype, and, when heterozygous, led to HNPP. Two patients with DSS had classic symptoms characteristic of this disease: from birth, they exhibited generalized muscle hypotonia and poor suckling, and delayed motor development was noted from the first months of life. On clinical examination, both siblings exhibited generalized limb muscle hypotonia, predominantly distal muscle atrophy, and muscle weakness.

We performed functional analysis of the identified variant c.179-2809A>G. The results of fibroblast RNA analysis and minigene assay clearly demonstrated that the c.179-2809A>G variant activates a 97 bp PE in intron 3, leading to formation of the premature stop codon and synthesis of severely shortened protein (Figure 4). Since haploinsufficiency is a known mechanism underlying HNPP, our functional analysis confirms that the c.179-2809A>G variant in the heterozygous state is sufficient to cause HNPP [10].

In the homozygous state, the c.179-2809A>G variant leads to a severe decrease in the level of correct mRNA isoform and is associated with the development of typical DSS. DSS is a rare hereditary disorder that belongs to the group of demyelinating neuropathies and represents a severe form of CMT [17]. Several genes, including *PMP22* (17p12), can be involved in the development of this syndrome. The mode of inheritance is most commonly autosomal dominant; autosomal recessive inheritance is less frequent [18]. According to the HGMD Professional 2025.1 database, to date, over 33 pathogenic variants in the *PMP22* gene have been identified as causative for DSS, of which only seven are associated with autosomal recessive inheritance. In cases of dominant inheritance, most variants are missense mutations that result from the production of a mutant protein that remains present in the cell, in contrast to cases involving *PMP22* deletion or duplication. The existence of different pathogenic mechanisms in case of missense mutations and *PMP22* deletions/duplications has been described by Anneke Gabreëls-Festen [18].

Of the 26 variants described with a dominant type of inheritance, only five are not missense variants. First, in three cases, the development of DSS with a dominant mode of inheritance was caused by a duplication of the *PMP22* gene in the heterozygous state, which is relatively uncommon [18]. This may be associated with the presence of certain modifying factors that we may not yet know about, or the presence of other pathogenic variants in the *PMP22* gene on a different allele that may have been missed during diagnosis. Anneke Gabreëls-Festen also notes in her study a pronounced phenotypic variability in families with *PMP22* duplication [18]. The second variant is a triplication of the *PMP22* gene (copy number *n* = 4), which leads to a severe CMT1 phenotype [9]. The third variant is a pathogenic 650 bp deletion, encompassing the miR-29a binding site in the 3′ untranslated region (3′-UTR), resulting in increased *PMP22* expression, which leads to a more severe phenotype than that observed in CMT1 [19]. The fourth variant is a deletion of exon 4 in the heterozygous state, which leads to a severe phenotype [20]. The development of a severe phenotype is most likely associated with the fact that skipping exon 4 does not lead to a frameshift and subsequent NMD. Accordingly, the altered protein remains in the cell, and the pathogenic mechanism of disease development differs from that observed in whole-gene deletions [20]. The fifth variant is a pathogenic variant, c.238_239del (p.(Leu80ValfsTer142)), which leads to a frameshift and, supposedly, produces a 49-amino-acid-longer peptide [21].

DSS patients with homozygous or compound heterozygous variants in the *PMP22* gene are rare. According to the HGMD Professional 2025.1, eight such cases have been reported (Table 2).

As can be seen from the table, only three cases involved variants that may lead to an absence of protein and were found on both alleles (cases 6, 7, 8). Patients 6 and 7 exhibited severe clinical symptoms similar to those observed in the siblings described in the present study. Patient 8 had less severe motor impairment compared to the other cases.

The number of reported cases with homozygous or compound heterozygous variants (only eight) is lower than expected, given the prevalence of CMT1A associated with a heterozygous duplication of *PMP22*, which occurs at a frequency of approximately 1 in 5000 [4]. As explained by Jun Li et al., the low number of homozygous or compound heterozygous variants may be due to the fact that complete absence of the PMP22 protein can lead to embryonic lethality. This is consistent with observations that homozygous *Pmp22* knockout mice (*Pmp22* −/−) have difficulty breeding, exhibiting a reproductive rate much lower than that of heterozygous *Pmp22* +/− mice [4]. At the same time, a single *PMP22*-null patient has been described whose motor impairments were less severe than those observed in the siblings reported in the present study [16]. These differences may represent phenotypic variability. But also, this difference in the severity of the phenotype of patients with *Pmp22* −/− and patients with other homozygous/compound heterozygous variants may be due to the fact that the mutant isoform, which does not undergo NMD in cases involving splice-affecting variants, expresses a mutant PMP22 protein that is unable to be transported from the endoplasmic reticulum to the plasma membrane and forms heterodimers with wild-type PMP22, resulting in protein aggregates. Additionally, missense variants of *PMP22* may potentially affect the secretion pathways of extracellular matrix protein components [27] and proper folding of the PMP22 protein. These mechanisms may potentially lead to a more severe phenotype than complete absence of PMP22. Such hypotheses may also explain the presence of more severe phenotypes in patients with missense variants and splice-affecting variants in a compound heterozygous state with a gross deletion of the *PMP22* gene [4].

Given that the c.179-2809A>G variant leads to haploinsufficiency, we hypothesized that this variant could be present in patients demonstrating a typical clinical picture of HNPP, but lacking the *PMP22* deletion. Subsequently, an additional family carrying this variant was identified, and the variant was found to segregate with the disease phenotype within that family. A limitation of this study is that NGS was not performed for this family; therefore, we cannot definitively conclude that this variant is the sole cause of HNPP in affected family members. Nevertheless, the genetic spectrum associated with typical HNPP is largely restricted to a limited number of genes.

Since the c.179-2809A>G variant was identified in two unrelated Avar families, it can be hypothesized that this variant is relatively common in the Avar group due to the founder effect. Unfortunately, in this work, we were unable to conduct a study of this variant in an Avar group in order to prove or disprove this assumption. Therefore, in cases of suspected demyelinating peripheral neuropathy among individuals of Avar origin, it is advisable not only to assess *PMP22* gene copy number, but also to screen specifically for this variant.

Although targeted therapies for hereditary diseases have been actively developed and implemented in recent years, there is currently no pathogenetic treatment available for any form of CMT. The most common therapeutic strategy focuses on suppressing *PMP22* overexpression resulting from an increased gene copy number. Suzan Boutary et al. developed an approach based on RNA interference using small interfering RNAs (siRNAs) targeting *PMP22*, which are conjugated with squalene (SQ) [28]. Hien Tran Zhao et al. developed an approach based on the suppression of *PMP22* mRNA expression using antisense oligonucleotides [29]. Benoit Gautier et al. proposed an approach using an adeno-associated virus (AAV2/9) to deliver a small hairpin RNA (shRNA) targeting *PMP22* mRNA [30]. Marina Stavrou et al. described a method involving the use of microRNA miR-871 and an adeno-associated virus (AAV9) [31]. Ji-Su Lee et al. used CRISPR/Cas9 to suppress *PMP22* gene expression by targeting the TATA box of the *PMP22* P1 promoter [32]. All of the approaches mentioned above are not suitable for the patients described in this study, as they are aimed at suppressing protein overexpression caused by *PMP22* gene duplication. In this study, we proposed a personalized gene therapy approach for the c.179-2809A>G variant, based on antisense oligonucleotides that modulate splicing (antisense splice-modulating oligonucleotides, ASMOs). The question of delivering ASMOs to Schwann cells remains a topic of debate. Both viral (adeno-associated viruses, herpes simplex viruses, lentiviruses) and non-viral delivery vectors (lipids, peptides, antibodies, squalene) are used. And despite the relatively successful study of these delivery methods, each of them has its own problems or limitations when used in vivo. Therefore, further research is essential to overcome existing challenges, including selective delivery, blood–brain barrier penetration, and distribution in the peripheral nervous system, as well as long-term efficacy and safety [33,34]. Regarding our research in the field of therapy, these are preliminary in vitro molecular findings, since correcting a splicing defect at the transcript level does not inherently prove that the therapy will alleviate symptoms in vivo. And further research is needed to imply clinical or symptomatic efficacy this approach.

## 4. Materials and Methods

### 4.1. Patients

Two families from Dagestan were examined. The first family included two siblings and their parents, who are not consanguineous and are of Avar origin. They underwent examination at the Research Centre for Medical Genetics (RCMG). The second family included the proband, siblings, and their parents, who are also non-consanguineous and of Avar origin. They were examined at the RCMG. Medical records, blood samples from all members of both families, and skin biopsies from all members of the first family were obtained. The available affected family members underwent NCS of peripheral nerves using Neuro-MVP-micro, Neurosoft (Ivanovo, Russia), and nerve ultrasound (US) using Hitachi Aloka ProSound F37 (Hitachi Aloka Medical Ltd., Tokyo, Japan). All patients gave written informed consent for publication of the data.

### 4.2. DNA Analysis

Molecular genetic analysis was carried out on genomic DNA samples extracted from peripheral blood lymphocytes of all examined individuals, according to the manufacturer’s protocol. *PMP22* copy number analysis was carried out using multiplex ligation-dependent probe amplification (MLPA) with the SALSA MLPA probemix P033 CMT1 (MRC-Holland, Amsterdam, The Netherlands).

Whole-genome sequencing (WGS) of the patients’ DNA was performed on a DNBSEQ-T7 genetic analyzer (MGI-Tech, Shenzhen, China) using paired-end 150 bp reads (PE150), provided by Biotech Campus LLC (Moscow, Russia). Library preparation was conducted using a PCR-free method with enzymatic fragmentation (MGI).

Segregation analysis of the identified pathogenic variant in the *PMP22* gene was carried out using Sanger sequencing on a 3130 xl Genetic Analyzer (Hitachi, Ibaraki, Japan) with the following specific primers: CTTCAGTCATAGATCATGTAAACC and CATACACCTAAAACAAGCAACAGTC.

Sequencing data processing was carried out using the “NGS-Data” information system (https://ngs-data-ccu.epigenetic.ru, accessed on 15 February 2026) and a “Neuropathies” virtual panel, which included 81 causative genes (Supplementary Table S1). Variants were named according to the Human Genome Variation Society (HGVS) nomenclature (http://www.hgvs.org/mutnomen/, accessed on 15 February 2026), using nucleotide and amino acid numbering based on published coding reference DNA sequences (*PMP22* NM_000304.4). All nucleotide sequence variants were compared with data published in the HGMD Professional 2025.1 (http://www.hgmd.cf.ac.uk/ac, accessed on 15 February 2026). The variant was classified by pathogenicity based on the criteria of the American College of Medical Genetics and Genomics (ACMG) guidelines (https://www.acmg.net/docs/standards_guidelines_for_the_interpretation_of_sequence_variants.pdf, accessed on 15 February 2026).

### 4.3. Preparation of Cultured Fibroblasts and Inhibition of Nonsense-Mediated Decay

Primary fibroblast cultures of patients from Family 1 were obtained from forearm skin biopsies and were grown in the proliferative medium “Amniokar” (PanEco Ltd., Moscow, Russia). For experiments, they were subcultured in ordinary growth medium “DMEM” supplemented with 15% fetal bovine serum (PanEco Ltd., Moscow, Russia). The fibroblast cultures are available in the Common Use Center “Biobank”, RCMG.

For NMD inhibition, fibroblasts were seeded in 6-well plates (350,000 per well). On the next day, cycloheximide (Cell Signaling Technology, Danvers, MA, USA) was added to a final concentration of 50 µg/mL, and cells were incubated for additional 2 days.

### 4.4. Minigene Assay

To create minigenes, the wild-type and the mutant fragments of the *PMP22* gene were cloned into the multiple cloning site of the pSpl3-Flu2-mTK vector [35]. The 3497 bp fragment (hg38: chr17:15,239,281–15,242,777) includes 260 bp of intronic sequence upstream of the predicted pseudoexon (PE), and the *PMP22* exon 4 with part of intron 4.

For transfection, HEK293T cells were seeded in 24-well plates. On the next day, cells were transfected with 500 ng of minigene plasmids and 1.5 µL of TurboFect Transfection Reagent (Thermo Fisher Scientific, Waltham, MA, USA). After 6 h, the medium was replaced with fresh one, and cells were incubated for additional 2 days before RNA extraction.

### 4.5. Antisense Vectors

Plasmids expressing modified U7 small nuclear RNAs (modU7snRNAs) were constructed as described previously [35]. Four modU7snRNAs with antisense sequence length of 25 bp were designed to target PE’s splice sites or splicing enhancer motifs (Table 3).

Lentiviral vectors were designed based on the transfer plasmid pULTRA (addgene #24129). The modU7snRNA cassettes in the reverse orientation were inserted into the PacI restriction site upstream of the UbC promoter using ClonExpress Ultra One Step Cloning Kit V3 (Vazyme, Nanjing, China). The same method was used to insert EGFP-P2A-PuroR cassette between AgeI and EcorI sites downstream of the UbC promoter (Supplementary Figure S1).

### 4.6. Lentivirus Preparation and Transduction

For lentivirus production, HEK293T cells were co-transfected in 6-well plates with 2.5 µg of plasmids at equimolar ratio: transfer plasmid, packaging plasmid psPAX2 (https://www.addgene.org/12260/, accessed on 15 February 2026), and envelope plasmid pCMV-VSV-G (https://www.addgene.org/8454/, accessed on 15 February 2026) using 7.5 µL of TurboFect Transfection Reagent (Thermo Fisher Scientific, Waltham, MA, USA). After 48 h, virus-containing medium was collected, filtered, and frozen at −80 °C. Virus aliquots then were used to determine the functional titer and to subsequently transduce fibroblasts at MOI~5 in the presence of 0.6 µg/mL of Polybrene Infection/Transfection Reagent (Sigma-Aldrich, St. Louis, MO, USA). On the next day, after transduction, puromycin (InvivoGen, San Diego, CA, USA) was added to final concentration of 2 µg/mL, and cells were incubated for additional 4 days.

### 4.7. RNA Analysis

RNA was isolated using FreeZol Reagent (Vazyme, Nanjing, China). cDNA was synthesized using HiScript III 1st Strand cDNA Synthesis Kit and oligo(dT) primers (Vazyme, Nanjing, China). The *PMP22* cDNA fragment spanning exons 3 and 4 was amplified by PCR with primers CACGATCGTCAGCCAATGGATC and CACTCATCACGCACAGACCAGC. The *SRSF3* cDNA fragment was amplified with primers CACTATGTGGCTGCCGTGTA and ATCGGGACGGCTTGTGATTT and was used as a control for NMD inhibition efficiency. Minigene-specific cDNA was amplified with primers ACAAAGAGACCTACGTCGAGCA and AGCTCGATCAGCACGGGCACGAT.

PCR products were Sanger sequenced and subjected to fragment analysis with primers labeled with 5′-end 6-FAM modification. The fragment analysis data was analyzed and visualized using Coffalyser.Net software (https://www.mrcholland.com/technology/software/coffalyser-net, accessed on 15 February 2026).

## 5. Conclusions

We were the first to describe and demonstrate the pathogenicity of the deep intronic variant c.179-2809A>G in the *PMP22* gene, which may be common in the Avar group. We also described the first reported case of Dejerine–Sottas syndrome associated with a homozygous deep intronic variant in the *PMP22* gene. We demonstrated the molecular genetic mechanism underlying the pathogenesis of DSS in affected individuals. Furthermore, we showed that this variant in the heterozygous state leads to the development of HNPP. In addition, we proposed a personalized gene therapy approach targeting the c.179-2809A>G variant.

## Figures and Tables

**Table 1:** Patients with HNPP. y—year, NCS—nerve conduction study, DML—distal motor latency, CV—conduction velocity, US—ultrasound, CSA—cross-sectional area.

Patient	1.1	1.2	1.3	2.1	2.2	2.3	2.4
Age (y)	44	38	14	57	24	21	11
Complaints	numbness and tingling in the upper and lower extremities	numbness and tingling in the upper and lower extremities	episodic numbness in hands	no	episodic numbness in hands	no	no
Episode of paresis Age of episode Site	no	no	no	yes 50 y right arm	yes 22 y right arm	no	no
Neurological status	lower leg and foot muscles atrophy, left hypothenar muscles mild hypotrophy, reduced tendon reflexes	foot muscles mild hypotrophy, reduced ankle reflexes	normal	reduced tendon reflexes	reduced tendon reflexes	reduced tendon reflexes	reduced tendon reflexes
NCS: ↑ medianus DML	yes	yes	no	yes	yes	no	yes
NCS: ↓ ulnaris CV at elbow	yes	yes	yes	yes	yes	yes	yes
NCS: ↓ peroneus CV at fibular head	yes	yes	no	yes	yes	yes	n/a
Nerve US: ↑ CSA at entrapment sites	yes	yes	yes	n/a	n/a	yes	n/a

**Table 2:** Cases of Dejerine–Sottas syndrome with autosomal recessive inheritance described in the literature. *PMP22*.

No	Allele 1		Allele 2		References
	c.DNA Position	Protein Change	c.DNA Position	Protein Change	
1	c.469C>T	p.(Arg157Trp)	del *PMP22*	no protein -	Xiaoxuan Liu et al., 2020 [22]
2	c.469C>G	p.(Arg157Gly)	del *PMP22*	no protein -	Chikahiko Numakura M.D. et al., 2000 [23]
3	c.469C>T	p.(Arg157Trp)	c.469C>T	p.(Arg157Trp)	Y. Parman et al., 1999 [24]
4	c.483A>G	p.(Ter161TrpexTer*10)	c.483A>G	p.(Ter161Trpext*10)	Alberto A. Zambon et al., 2020 [15]
5	c.320-1G>A	p.?	del *PMP22*	no protein	Xiaoxuan Liu et al., 2020 [22]
6	del ex 2-3	p.?	del *PMP22*	no protein	Khalid Al-Thihli et al., 2008 [25]
7	del ex 5	p.?	del *PMP22*	no protein	Akiko Abe et al., 2010 [26]
8	del *PMP22*	no protein	del *PMP22*	no protein	Mario Andre Saporta et al., 2010 [16]

**Table 3:** The modU7snRNAs sequences.

Name	Target	Antisense	Length
U7.1	CAGTCATAGATCATGTAAACCAGAA	TTCTGGTTTACATGATCTATGACTG	25
U7.2	CCAAATCTTTCCTCTATGAAACAAC	GTTGTTTCATAGAGGAAAGATTTGG	25
U7.3	CAACTCTCTGGCCACTTTTATCCTT	AAGGATAAAAGTGGCCAGAGAGTTG	25
U7.4	GGCCACTTTTATCCTTTCAACGTAA	GTTGAAAGGATAAAAGTGGCC	25

## Data Availability

Data are contained within the article.

## Supplementary Materials

The following supporting information can be downloaded at: https://www.mdpi.com/article/10.3390/ijms27083572/s1.
