# Supplementary figures and images for: Identification and Targeted Correction of a Pathogenic PMP22 Deep Intronic Variant
Source: Int J Mol Sci. 2026 Apr 16;27(8):3572. doi: 10.3390/ijms27083572 (PMC13115777; doi:10.3390/ijms27083572)

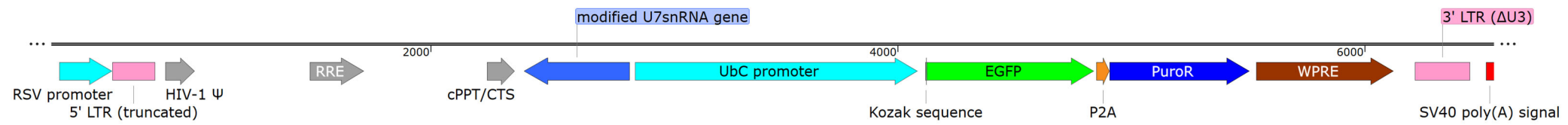

**Figure S1.** The structure of the lentiviral vector expression cassette)

Supplement: Supplementary file 1 [file ijms-27-03572-s001.zip › Supplementary Figure S1.pdf]
